# Subclinical psychotic experiences and subsequent contact with mental health services

**DOI:** 10.1192/bjpo.bp.117.004689

**Published:** 2017-03-07

**Authors:** Vishal Bhavsar, James H. Maccabe, Stephani L. Hatch, Matthew Hotopf, Jane Boydell, Philip McGuire

**Affiliations:** Vishal Bhavsar, MRCPsych, Department of Psychosis Studies, Institute of Psychiatry, Psychology and Neuroscience, King’s College London, London, UK; **James H. Maccabe**, PhD, Department of Psychosis Studies, Institute of Psychiatry, Psychology and Neuroscience, King’s College London, London, UK; **Stephani L. Hatch**, PhD, Department of Psychological Medicine, Institute of Psychiatry, Psychology and Neuroscience, King’s College London, London, UK; South London and Maudsley NHS Foundation Trust, London, UK; **Matthew Hotopf**, PhD, Department of Psychological Medicine, Institute of Psychiatry, Psychology and Neuroscience, King’s College London, London, UK; South London and Maudsley NHS Foundation Trust, London, UK; **Jane Boydell**, PhD, Cornwall Partnership NHS Foundation Trust, Bodmin, UK; **Philip McGuire**, PhD, Department of Psychosis Studies, Institute of Psychiatry, Psychology and Neuroscience, King’s College London, London, UK

## Abstract

**Background:**

Although psychotic experiences in people without diagnosed mental health problems are associated with mental health service use, few studies have assessed this prospectively or measured service use by real-world clinical data.

**Aims:**

To describe and investigate the association between psychotic experiences and later mental health service use, and to assess the role of symptoms of common mental health disorders in this association.

**Method:**

We linked a representative survey of south-east London (SELCoH-1, *n*=1698) with health records from the local mental healthcare provider. Cox regression estimated the association of PEs with rate of mental health service use.

**Results:**

After adjustments, psychotic experiences were associated with a 1.75-fold increase in the rate of subsequent mental health service use (hazard ratio (HR) 1.75, 95% CI 1.03–2.97) compared with those without PEs. Participants with PEs experienced longer care episodes compared with those without.

**Conclusions:**

Psychotic experiences in the general population are important predictors of public mental health need, aside from their relevance for psychoses. We found psychotic experiences to be associated with later mental health service use, after accounting for sociodemographic confounders and concurrent psychopathology.

**Declaration of interest:**

None.

**Copyright and usage:**

© The Royal College of Psychiatrists 2017. This is an open access article distributed under the terms of the Creative Commons Attribution (CC BY) license.

Psychotic experiences, such as hallucinations, thought interference and paranoid ideas, are common in the general population. However, they are only severe and/or frequent enough to reach diagnostic thresholds for schizophrenia or related psychotic disorders in a minority.^1^ Although people who report psychotic experiences in unselected, population-based samples have elevated risk for psychosis relative to people who do not,^2^ the absolute increase in this risk is low, due to the low incidence of psychotic disorders overall.^3^

If psychotic experiences are to be useful in predicting, and possibly averting, the later onset of clinical disorders, a key question is whether, and to what extent, these experiences are specifically associated with psychotic disorders or are associated with psychiatric disorders more generally.^4^ This issue is critically relevant to the current debate about whether early detection mental health services should be targeted at reducing the risk of a later psychotic disorder (e.g. in the context of the at-risk mental state (ARMS))^5^ or offer broader interventions aimed at reducing the risk of a range of mental health disorders.^6^^,^^7^

Although previous research has demonstrated consistent associations between psychotic experiences and use of mental health services for mental health problems such as anxiety, depression and post-traumatic stress disorder (PTSD), no studies to date have characterised this association by real-time clinical data, with objective, as opposed to self-reported, measures of service use. Furthermore, the extent to which symptoms of common mental health disorders in non-clinical individuals with psychotic experiences (such as depressive or anxiety or post-traumatic stress (PTS) symptoms) contribute to the later use of mental health services is unclear.

The first aim of this study was to quantify the association between psychotic experiences, ascertained in a representative community survey of South London, and subsequent use of local secondary mental healthcare services ascertained directly from electronic health records, over a period of 5 years follow-up. A secondary aim was to evaluate the role of symptoms of common mental health disorders as an alternative explanation for any association. Our main hypothesis was that psychotic experiences would be associated with a higher rate of later mental health service use. We also hypothesised that this association would persist after adjusting for the influence of symptoms of depression, anxiety and PTS.

## Method

### Sample details

This study linked a cross-sectional community health survey with a mental healthcare provider database. The South East London Community Health Study (SELCoH)^8^ is a representative household survey whose first wave (SELCoH-1) took place in 2008–2010. The survey used random household sampling to identify a representative sample of adults aged 16–90 years living in Lambeth and Southwark. The sampling was clustered by household, with all adults living in selected households invited to participate. Full details of the study, its sampling methods and representativeness are published.^8^ Among the 1698 participants surveyed, 86% gave permission for linkage to secondary mental health records, where those records were available. SELCoH-1 participants were therefore classified into two groups, based on whether or not linked records were found. Use of mental health services both before and after SELCoH-1 interview was collected. Data from SELCoH phase 2, a survey performed in 2012–2013 based on the same sample, were used to ascertain which individuals had left the catchment area or died.^9^ The SELCoH-1 study received approval from the King’s College London Research Ethics Committee, reference no. CREC/07/08-152. The Clinical Record Interactive Search (CRIS) data resource received ethical approval as an anonymised data set for secondary analyses from Oxfordshire REC C, reference no. 08/H0606/71+5.

### Measures

#### Psychotic experiences

The Psychosis Screening Questionnaire (PSQ)^10^ was used to assess psychotic experiences. This is a five-item questionnaire that assesses different psychotic symptom domains experienced in the previous year. These comprise the following: hypomania, strange experiences, paranoia, hallucinations and thought disorder. Each domain contains an initial ‘probe’ item, which is followed by secondary questions. Because this study was focused on non-affective psychotic symptoms, responses to the hypomania item were not examined. Individuals were considered to have psychotic experiences if they endorsed one or more secondary items in the four remaining domains. The Psychosis Screening Questionnaire displays good correspondence with psychosis items on the Schedules for Clinical Assessment in Neuropsychiatry^10^ and has seen frequent use in population studies.^11^

#### Covariates

SELCoH-1 collected sociodemographic, environmental and health information. For this analysis, age was grouped into 10-year categories. Ethnicity was operationalised as a five-category variable comprising White, Black African, Black Caribbean, Asian and other groups. Participants’ employment status was categorised into employed, student, unemployed and other. Highest educational attainment was categorised into ‘no qualifications’, ‘GCSE/O-level’, ‘A-Level’ and ‘degree level and above’. PTS symptoms were assessed by the PC-PTSD,^12^ a screening tool for PTSD designed for primary care use, which is based on the diagnostic criteria for PTSD in DSM-IV. The Clinical Interview Schedule (Revised, CIS-R)^13^ was used to measure symptoms of depressive and anxiety disorders in the form of a numerical score.

#### Use of mental health services

Data on mental healthcare utilisation for SELCoH-1 participants were derived from the National Institute of Health Research Biomedical Research Centre at the Maudsley’s Clinical Record Interactive Search.^14^ The South London and Maudsley NHS Foundation Trust (SLaM) is the sole provider of mental healthcare in the two boroughs of South London that were surveyed in SELCoH-1, covering a catchment population of approximately 0.62 million. The Trust has used a single electronic health record across all clinical services known as the electronic Patient Journey System (ePJS) since 2006, with more limited information available from 2001. CRIS extracts de-identified clinical data from the ePJS including structured fields for ICD-10 diagnoses, treatments and admissions to hospital. Linkage of SELCoH-1 to CRIS was carried by an independent in-house informatics team, the SLaM Clinical Data Linkage Service (CDLS), and used personal identifiers (name, date of birth, NHS number, postcode and gender) to probabilistically link survey data with matching electronic health records.^15^ Data on SELCoH-1 participants who had consented to record linkage were then scrutinised in CRIS. Information was available on date and route of referral to mental health services and ICD-10 diagnosis. Diagnoses in SLaM are generally arrived at through multidisciplinary discussion among the team primarily responsible for the patient.^14^ Primary diagnoses were grouped into common mental disorders, serious mental illness, other diagnoses and patients in whom no diagnosis was made. Durations of separate care episodes for each individual were added together to derive a variable reflecting total duration of mental health service use (grouped into total care episodes of durations between 0 and 100 days, between 101 and 1000 days, and 1001 days or above). A categorical variable on referral source contained groups for referral via accident and emergency departments, self-referral/referral from school/referral from a carer, referral by a general practitioner, a non-psychiatric clinical specialty, police/medium secure mental health services, social services, other services and a category for participants where the source of referral was missing or recorded as null.

### Analysis

All analyses were carried out in STATA 14^16^ and took account of household non-response and clustering of respondents within households, by inverse probability weights and survey commands in STATA. The estimation of non-response weights is further described in Hatch *et al*.^8^ Counts/percentages for categorical variables were described for those who made contact with mental health services and those who did not. Categorical distributions for linked participants, for diagnosis, care duration and referral source were described by psychotic experience status and evaluated by chi-squared tests. For Cox regression, only use of mental health services commencing after the survey interview was analysed; time was defined as elapsed time since birth and observation period as time elapsed from SELCoH-1 interview. Conclusion of follow-up time was defined as removal from the population at risk, either due to the occurrence of the outcome (use of mental health services), due to coming to the end of follow-up time (15 May 2015) or due to being untraceable or dead at date of follow-up interview. Rates stratified by psychotic experience status, age, gender and the other sociodemographic and clinical variables were calculated. Proportionality of hazards by psychotic experience status was assessed graphically by log–log plots and a test for the null hypothesis of a zero slope in the log hazard ratio (HR) function, by Schoenfeld residuals. Covariates for Cox regression were evaluated for inclusion based on the change in estimates method – covariates for which the adjusted association changed by more than 10% from the crude association were retained for inclusion in the fully adjusted model.^17^ Partially and fully adjusted rate ratios were estimated for secondary mental health service use.

## Results

### Categorical description of linkage and linked participants

Of 1698 survey participants, 243 individuals did not consent to linkage: information on whether or not these individuals used mental healthcare was therefore not available. These records were dropped from the analysis. Chi-squared comparison of those who did with those who did not consent to linkage indicated that there were no significant differences in gender, educational attainment or employment status between the two groups. However, consenting participants were more likely to be older and of White ethnicity (data available on request from authors). The overall proportion of individuals using secondary mental health services at any time was 12.10%. Nearly 40% of people who used secondary mental health services reported psychotic experiences at baseline compared with around 17% of people who did not use these services (Table 1). Of those using secondary mental health services, 64% were female compared with 55% of those who did not. Participants using secondary mental health services had lower educational attainment, were more likely to be unemployed, and were more likely to report symptoms of common mental health disorders.

**Table 1:** Counts (column percentages in brackets) and crude rates of mental health service use for psychotic experiences and selected SELCoH-1 variables

	Did not use secondary mental health services	Used secondary mental health services	Rate (contacts per 1000 person-years)	95% CI
Psychotic experiences				
No	1060 (82.88)	108 (61.36)	9.58	7.42 to 12.56
Yes	214 (16.73)	67 (38.07)	19.48	13.14 to 30.00
Missing	5 (0.39)	1 (0.57)		
Gender				
Male	570 (44.57)	63 (35.80)	7.97	5.43 to 12.18
Female	709 (55.43)	113 (64.20)	14.25	11.04 to 19.2
Missing	0 (0.00)	0 (0.00)		
Age				
16–24	269 (21.03)	31 (17.61)	11.02	6.81 to 18.94
25–34	296 (23.14)	26 (14.77)	5.67	2.99 to 12.11
35–44	264 (20.64)	32 (18.18)	9.58	5.74 to 17.16
45–54	198 (15.48)	36 (20.45)	8.92	4.87 to 18.08
55–64	126 (9.85)	23 (13.07)	14.70	8.31 to 28.43
65+	126 (9.85)	28 (15.91)	29.51	19.57 to 46.27
Missing	0 (0.0)	0 (0.0)		
Employment				
Employed	743 (58.09)	41 (23.30)	5.71	3.83 to 8.90
Students	187 (14.62)	22 (12.50)	10.67	5.98 to 20.86
Unemployed	107 (8.37)	37 (21.02)	23.12	13.71 to 41.64
Other	237 (18.53)	75 (42.61)	23.52	16.83 to 33.76
Missing	5 (0.39)	1 (0.57)		
Education				
No qualifications	153 (11.96)	43 (24.43)	25.07	16.77 to 38.98
GCSE	247 (19.31)	52 (29.55)	14.16	9.22 to 22.82
A-level	317 (24.78)	37 (21.02)	10.88	6.99 to 17.83
Degree level	553 (43.24)	38 (21.59)	5.94	3.79 to 9.86
Missing	9 (0.70)	6 (3.41)		
Ethnicity				
White	814 (63.64)	120 (68.18)	11.52	8.88 to 15.2
Black Caribbean	98 (7.66)	18 (10.23)	14.41	7.24 to 32.59
Black African	174 (13.60)	13 (7.39)	5.93	2.49 to 17.79
Asian	43 (3.36)	5 (2.84)	11.70	3.61 to 56.60
Other	148 (11.57)	20 (11.36)	13.45	7.56 to 26.17
Missing	2 (0.16)	0 (0.00)		
Post-traumatic stress symptoms				
None	1219 (95.31)	147 (83.52)	10.47	8.34 to 13.31
Possible case	48 (3.75)	27 (15.34)	29.89	15.21 to 66.03
Missing	12 (0.94)	2 (1.14)		
Symptoms of common mental health disorder				
No	1010 (78.97)	92 (52.27)	7.79	5.84 to 10.61
Yes	268 (20.95)	82 (46.59)	23.79	17.18 to 33.77
Missing	1 (0.08)	2 (1.14)		
Total	1279 (100)	176 (100)	11.24	9.07 to 14.10

### Use of mental health services

In participants with any mental health service use, psychotic experiences were not statistically associated with either diagnoses or referral source. However, a greater proportion of those who reported psychotic experiences had care episodes totalling 1000 days or more in duration, compared with those who did not report psychotic experiences (Table 2). Among 15 individuals who used mental health services who were assigned the diagnostic grouping of serious mental illness, 12 were recorded as having psychotic disorders and 3 were diagnosed with bipolar affective disorder.

**Table 2:** Service use information on SELCoH-1 participants who used secondary mental healthcare, by psychotic experience (PE) status (*n*=175)

	No PEs (%)	PEs (%)	Chi-squared *P*-value (d.f.)
Diagnostic group			
CMD	37 (34.26)	28 (41.79)	
SMI	8 (7.41)	7 (10.45)	
Dementia	9 (8.33)	2 (2.99)	
Other	18 (16.67)	8 (11.94)	
Null	36 (33.33)	22 (32.84)	0.465 (4)
Total duration of contact within mental healthcare			
Less than 100 days	49 (45.37)	20 (29.85)	
100 to less than 1000 days	45 (41.67)	29 (43.28)	
1000 days or more	14 (12.96)	18 (26.87)	0.031 (2)
Referral source			
Emergency department	11 (10.19)	9 (13.43)	
Self/school/carer	5 (4.63)	5 (7.46)	
GP	38 (35.19)	25 (37.31)	
Null	8 (7.41)	1 (1.49)	
Other	25 (23.15)	20 (29.85)	
Other specialty	16 (14.81)	6 (8.96)	
Police/MSU	1 (0.93)	1 (1.49)	
Social services	4 (3.70)	0 (0.0)	0.720 (7)
Total	108 (100)	67 (100)	

CMD, Common mental disorders; SMI, severe mental illness; MSU, medium secure unit.

### Survival analysis of time to first mental health service use

#### Description of survival data

Of 1455 survey participants consenting to linkage of their survey data with electronic health records, person-time for 94 individuals was excluded as follow-up had concluded before the baseline assessment interview (i.e. they used mental health services before the SELCoH-1 interview). Remaining were 1361 participants, among whom there were 82 individuals who used mental health services after interview. Of the remaining participants, 12 died after the survey interview, and 105 were found to be untraceable at follow-up interview and considered to have been censored at the date that contact was attempted. The total follow-up time in the whole sample was 6926.28 years. Per individual, the median follow-up time was 5.25 years. Among participants without psychotic experiences at survey interview, median follow-up time was 5.27 years, and in those with psychotic experiences, it was 5.20 years. We found no statistical evidence for a difference in follow-up times between those with and without psychotic experiences, based on a Wilcoxon rank-sum test (*P*=0.522). Follow-up time did not vary significantly by gender or other sociodemographic variables analysed in this study (data available on request from authors).

The overall rate of mental health service use in the sample population was around 11 contacts per 1000 person-years of follow-up (rate=11.24, 95% CI 9.07 to 14.10; Table 1). People who reported psychotic experiences at survey interview had around twice the crude rate of subsequent mental health service use compared with those who did not. A higher rate of mental health service use was associated with female gender, lower educational attainment, unemployed status and baseline symptoms of depression, anxiety and PTSD (Table 1).

#### Cox regression analysis

**Fig. 1:**
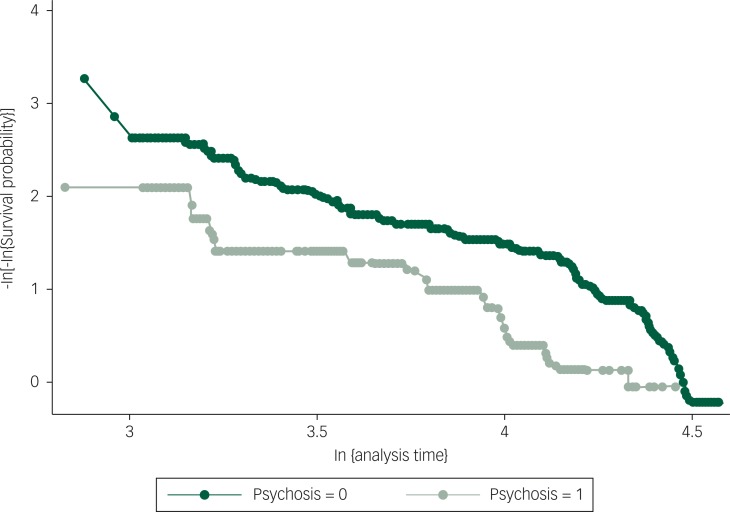
Approximately parallel curves for psychotic experiences and no psychotic experiences on axes of –log[log(survival probability)] against log(analysis time), implying that proportionality of hazards has not been violated. Global test of proportional hazards assumption for the final regression model, based on Schoenfeld residuals, was 0.516.

Among the 1361 participant records providing follow-up information, 24 individuals had missing data on one or more of the modelled variables and were removed from the analysis, leaving a final analytic sample for multivariate modelling of 1337. Graphical and inferential methods did not indicate that proportional hazards assumptions had been violated (*P*-value from the formal test of the proportional hazards assumption, based on Schoenfeld residuals, was 0.5164, see Fig. 1). In multivariate Cox regression, psychotic experiences were associated with a twofold increase in the rate of mental health service use, after adjusting for age, gender, employment status, educational attainment and ethnic group (HR=1.91, 95% CI 1.13 to 3.26). This association was partially attenuated on inclusion of symptoms of depression and anxiety, and this association was further reduced by the addition of PTS symptoms. Nevertheless, good statistical evidence remained for an association between reporting psychotic experiences and time to mental health service use, after accounting for these variables (HR=1.75, 95% CI 1.03 to 2.97; Table 3).

**Table 3:** Multivariate Cox regression models presenting hazard ratios (HRs) for the effect of psychotic experiences on the hazard of contact with mental health services

	HR comparing psychotic experiences with no psychotic experiences	95% CI	*P*
Adjusted for age only	2.41	1.48 to 3.92	<0.001
Adjusted for age and gender	2.39	1.46 to 3.92	0.001
Adjusted for age, gender and educational attainment	2.13	1.28 to 3.55	0.004
Adjusted for age, gender, educational attainment and employment	1.87	1.11 to 3.17	0.02
Adjusted for age, gender, educational attainment, employment and ethnic group	1.91	1.13 to 3.26	0.016
Adjusted for age, gender, educational attainment, employment, ethnic group and symptoms of common mental health disorders	1.90	1.12 to 3.22	0.017
Adjusted for age, gender, educational attainment, employment, ethnic group, symptoms of common mental health disorders and PTS symptoms	1.75	1.03 to 2.97	0.039

All models based on follow-up data from 1337 participants.

## Discussion

### Summary of findings

This investigation found that in a representative population sample, psychotic experiences were prospectively associated with approximately a doubling in the likelihood that they would use secondary mental health services over the next 5 years. This effect was partially, but not completely, accounted for by concurrent symptoms of depression, anxiety and PTS at baseline. Survey participants who had reported psychotic experiences spent longer under the care of mental health services than those who had not reported psychotic experiences.

### What is already known on this topic

Psychotic experiences are distressing,^18^ and a number of studies have reported an association between psychotic experiences and use of mental health services, in both community^19^ and clinical high-risk samples.^6^ One study did not find an association^20^ – this study investigated a psychiatric out-patient group, comparing those who returned following an initial assessment with those who did not.

Previous studies examining service use in individuals reporting psychotic experiences have not measured service use subsequent to their assessment or attempted to estimate the effect of psychotic experiences on service use duration. Armando *et al*^21^ reported a strong correlation between subtypes of psychotic experiences and use of mental health services, but it is unclear what proportion of people with any psychotic experiences reported service use. Devylder *et al*^22^ analysed data from the Collaborative Psychiatric Epidemiology Survey to investigate the use of mental health services in people with and without psychotic experiences and found that nearly 60% of people with psychotic experiences reported lifetime mental health service use, compared with around 40% of people without. In an analysis of the Adult Psychiatric Morbidity survey 2007, Murphy *et al*^19^ reported elevated risks of lifetime use of mental health services in people with psychotic experiences, with paranoid experiences associated with a more than twofold increase in risk of reporting contact with mental health services in the previous year. However, all of these studies relied on self-reported service use,^19^^,^^21^^–^^23^ which could be vulnerable to recall bias;^24^ for example, individuals who have more extensive mental health service use and may have the most severe disorders, or may have better recall of service use than individuals with more transient distress.^25^

Overall, the previous literature suggests that psychotic experiences are associated with an increased likelihood of using mental health services, with more limited evidence that this association remains after accounting for possible confounding effects of other mental health symptoms. However, the assessment of mental health service use has usually relied on self-report rather than directly measured mental health service use, and no studies to our knowledge have assessed the association between psychotic experiences and mental health service use prospectively.

### What this study adds

Our findings extend this literature by suggesting that psychotic experiences significantly increase the likelihood of subsequent use of mental health services. Moreover, this association was evident in a prospective follow-up of a large sample gathered via a representative household survey. Service use was measured by directly analysing mental health records from the comprehensive mental healthcare provider for the local population, and the association was not simply attributable to the influence of concurrent symptoms of common mental health disorders.

### Explanations for the association

Psychotic experiences could be associated with subsequent service use because they are concurrent with other mental health symptoms or conditions. Associations have been documented between psychotic experiences and symptoms of depression,^18^^,^^26^ anxiety,^27^^,^^28^ obsessive–compulsive disorder,^29^ PTSD,^30^^,^^31^ as well as suicidal behaviours.^32^^,^^33^ However, we found that the effect of psychotic experiences on subsequent service use persisted after adjusting for the presence of depression, anxiety and PTS symptoms.

### Importance

Our results suggest that psychotic experiences in the general population are a significant predictor of a need for later mental health service use, after accounting for the correlation between psychotic experiences and symptoms of common mental health disorders. At present, clinical care for psychotic experiences is limited to the subgroup of people who have psychotic experiences that occur as part of an at-risk mental state.^5^ Moreover, these services are focused on reducing the risk of psychotic disorders, as opposed to mental disorders in general. Our findings indicate that the presence of psychotic experiences is associated with an increase in later mental health service use at the level of the general population. This service use may involve a range of mental disorders rather than being limited to psychosis.^4^^,^^7^

### Strengths and limitations

One limitation of the study was that we did not have information about how long psychotic experiences had been present before the baseline assessment, which precluded analysis of the time between the onset of psychotic experiences and subsequent use of mental health services. In addition, diagnostic information on those who used mental health services was incomplete and based on routine clinical recordings, rather than validated diagnostic measures. Although the outcome was defined on the basis of routine clinical data, thereby limiting comparison with formal epidemiological designs, the results indicate that psychotic experiences are related to subsequent mental healthcare need at the population level, irrespective of diagnosis. In this analysis, concurrent mental health symptoms were adjusted for as potential confounders. However, the relationship between different mental health symptoms and psychotic experiences could be more complex. For example, it was not possible to determine whether depression/anxiety symptoms are confounders of the association between psychotic experiences and service use, or whether they are mediators. Finally, this analysis made the assumption that those participants who did not make contact with mental health services and who were not found to be untraceable at follow-up interview remained at risk for the outcome over the duration of the study. We may therefore have overestimated the length of time people were at risk for the outcome in this study, underestimating the overall rate of mental health service use. However, we consider this to be a conservative measure and that any bias introduced by this was probably in the null direction. Furthermore, people moving out of the study area might be expected to be more resilient/resourceful/mobile and therefore be less likely to seek mental healthcare in the context of psychotic experiences compared with those without psychotic experiences; however, we feel this is unlikely as a mechanism of bias in this study – we expect that most participants moved out of the study area because they were displaced by urban development and regeneration and not because of outward migration of more resilient participants.

### Concluding remarks

In general population-based sample of south-east London, psychotic experiences were associated with later use of mental health services over a period of 5 years of follow-up. This subsequent mental health service use was related to mental health problems in general, rather than specifically to psychotic disorders.
